# Diagnostic Accuracy of Computed Tomography Scout Film and Chest X-ray for Detection of Rib Fractures in Patients with Chest Trauma: A Cross-sectional Study

**DOI:** 10.7759/cureus.3875

**Published:** 2019-01-13

**Authors:** Muhammad Awais, Basit Salam, Naila Nadeem, Abdul Rehman, Noor U Baloch

**Affiliations:** 1 Radiology, The Aga Khan University, Karachi, PAK; 2 Internal Medicine, Rutgers New Jersey Medical School, Newark, USA

**Keywords:** rib fracture, scout film, computed tomography, plain radiograph, trauma

## Abstract

Background: Rib fractures are a major source of morbidity in patients with chest trauma. Computed tomography (CT) scout film is a low-dose image that is obtained prior to a complete chest CT study for all patients undergoing a CT scan. In this study, we evaluated the diagnostic performance of CT scout film vis-à-vis that of chest X-ray for detection of rib fractures using chest CT scan as the reference standard.

Methods: A cross-sectional study was performed at the radiology department of Aga Khan University Hospital (Karachi, Pakistan) from October 1, 2013 to September 31, 2014. Patients who underwent CT chest for evaluation of thoracic trauma were included in the study. Sensitivity and specificity of chest X-ray and CT scout film were calculated.

Results: A total of 207 patients were included in the study (193 were male). Penetrating and blunt thoracic injuries affected 104 (50.2%) and 103 (49.8%) patients respectively. On CT chest, 75 (36.2%) patients had evidence of rib fractures. Sensitivity and specificity of CT scout film for detection of rib fractures were 56% and 87.9%, while those of chest X-ray were 61.3% and 98.5% respectively. The overall accuracy of CT scout film and chest X-ray for detection of rib fractures were 76.3% and 85% respectively.

Conclusion: Diagnostic performance of CT scout film for detection of rib fractures was comparable to that of the plain chest radiograph. CT scout film does not provide any additional information or advantage over a plain chest radiograph. In patients with severe thoracic trauma, CT chest remains the modality of choice for accurate delineation of rib fractures and associated internal injuries.

## Introduction

Thoracic trauma constitutes up to 15% of all cases of trauma cases and has been reported to have a mortality rate of up to 25% [1]. As much as 70% of thoracic trauma occurs by blunt mechanisms, while penetrating injuries account for the rest of 30% [2]. Rib fractures mostly occur as a consequence of blunt injuries to the chest, although firearm injuries, which cause penetrating trauma to the chest, can rarely result in rib fractures as well [3]. On plain radiographs and computed tomography (CT), these fractures are commonly recognized as a discontinuity or disruption of the cortical outline of the bony rib [4]. Blunt and penetrating thoracic injuries can result in pneumothorax, hemothorax, lung contusions, and rib fractures. Rib fractures have a special significance as they can contribute to significant morbidity, especially in elderly patients with multiple co-morbidities [5]. Lower rib fractures may raise suspicion for intra-abdominal visceral injury, while fractures of the first rib imply high momentum injuries and warrant evaluation for injuries to the heart and great vessels [6].

Diagnosis of rib fractures is easily made on thoracic CT scans [4]. However, in patients presenting with isolated blunt trauma to the chest of a minor intensity, CT scans are usually not ordered. This is because CT scans have a radiation dose that is at least seventy times higher than that of plain chest radiographs [7]. Moreover, CT chest may also impose a substantial economic burden, especially for patients treated at smaller centers of lower- to middle-income countries [8]. When CT scans are not ordered in such cases, most clinicians rely on physical examination and plain radiographs for clinical decision making. Previous studies suggest that chest radiographs may be less sensitive for detection of rib fractures, especially of the lower ribs [9-10]. The implications of missed rib fractures in such patients may be serious and can potentially lead to delayed diagnosis of serious injuries [11].

CT scout film is a tool that has been mainly used by radiographers for determining the area to be scanned in a CT study [12]. This has been mostly ignored by radiologists in their regular reporting until recently. Over the past few years, studies have explored the use of CT scout films for various purposes [13]. We hypothesized that CT scout films may offer good sensitivity and specificity for detection of rib fractures. To test this hypothesis, we performed a cross-sectional study at our institution and evaluated the diagnostic performance of CT scout film and plain chest X-ray for the detection of rib fractures in patients presenting with thoracic trauma.

## Materials and methods

A cross-sectional study was performed at the department of radiology of Aga Khan University Hospital (Karachi, Pakistan). Ethical approval from the institutional ethics review committee was sought prior to the initiation of this study. A pilot study was conducted at the Aga Khan University Hospital, which reported a sensitivity of 83% and specificity of 100% for detection of rib fractures on scout film using complete CT scan as the reference standard. A previous study published from Pakistan had reported that the prevalence of rib fractures in patients presenting with thoracic trauma was 87% [14]. Using these values of sensitivity, specificity, and prevalence with desired precision at 0.05% and confidence interval of 95%, the required sample size for this study was calculated to be 176. We used a non-probability, consecutive sampling strategy to include patients in this study. Individuals aged 18 years or more of either gender, who presented to the emergency department (ED) of our institution within twelve hours of thoracic trauma, were eligible for inclusion in the study, provided they were referred to our department for a chest CT scan (i.e., axial sections with coronal and sagittal reformations) by the primary emergency physician. Informed consent was taken from all the subjects or their next of the kin in cases where the patients were either unconscious or having altered mental status. Patients were excluded from the study if they: (a) presented more than twelve hours after thoracic trauma; (b) did not give informed consent; (c) had prior history of fractured ribs or severe thoracic trauma; (d) had history of metastases involving the ribs; or (e) had a history of metabolic bone diseases.

Chest CT scans were performed using a 640-slice multi-detector computed tomography (MDCT) scanner (Aquilion ONE®; Toshiba Medical Systems, Japan). CT scout films were obtained in antero-posterior and lateral projections, which were then used to determine the area required for subsequent scanning. For the purpose of this study, CT scout films (frontal projection only) and chest CT scans were interpreted by a consultant radiologist having at least five years of experience in CT imaging. Additionally, reports of plain chest radiographs were retrieved from radiology information system for all patients. CT scout films and chest CT scans were meticulously evaluated for the presence of rib fractures and data was recorded in a structured pro forma. A complete chest CT scan was used as reference standard for detection of rib fractures. In order to minimize any potential bias, CT scout films were interpreted after an interval of at least one week following the interpretation of chest CT scans. Moreover, in order to minimize bias, the consultant radiologist was blinded to the results of the chest CT scan at the time of interpreting CT scout films.

**Figure 1:**
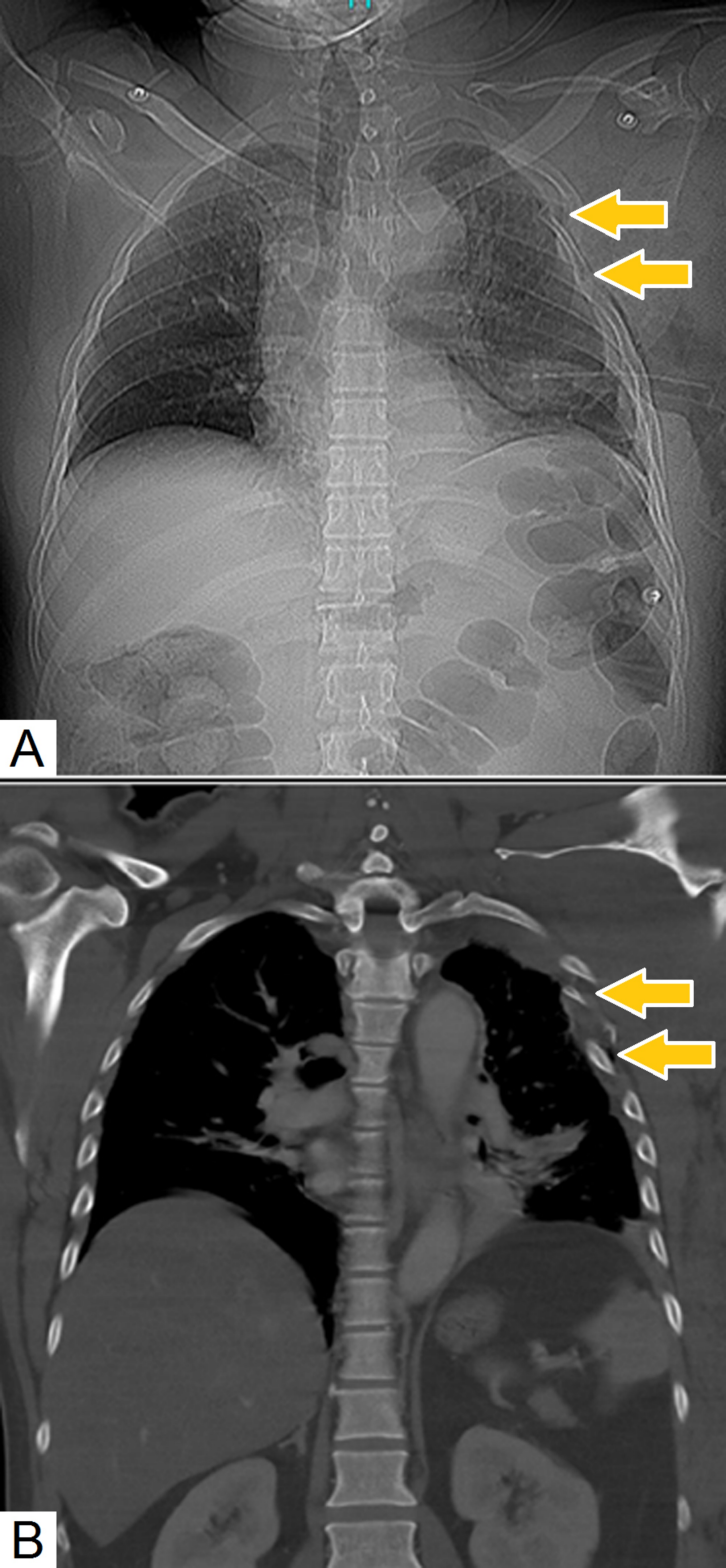
Comparison of scout film and thoracic computed tomography (A) Scout film demonstrating multiple left-sided rib fractures (arrows). (B) Coronal chest computed tomography (CT) images confirm the presence of multiple left-sided rib fractures (arrows).

**Figure 2:**
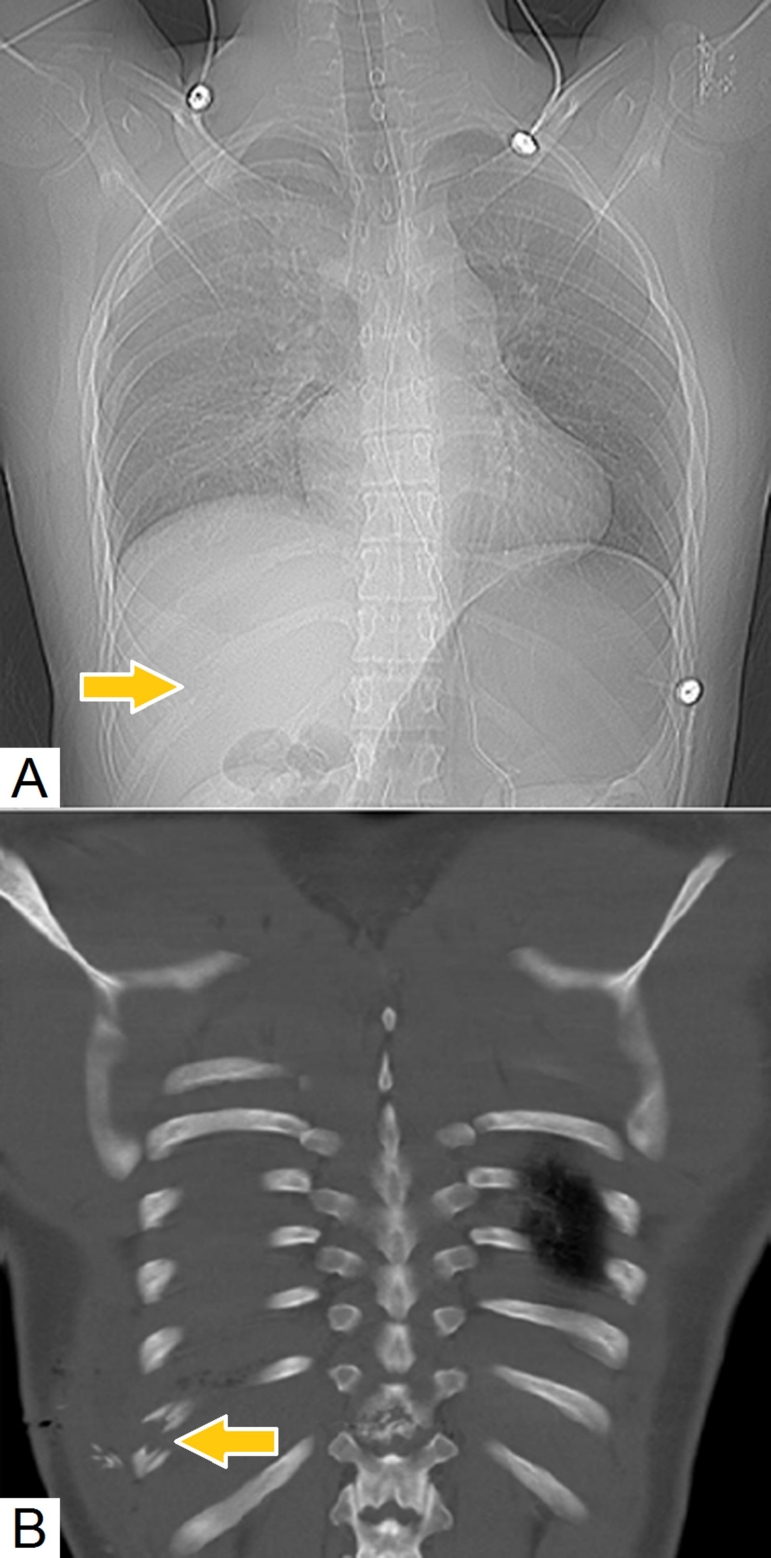
Depiction of rib fracture on scout film and thoracic computed tomography (A) Scout film of computed tomography (CT) depicting fracture of the right eleventh rib (arrow). (B) Thoracic CT in “bone” window settings confirm a fracture of the right eleventh rib (arrow).

Statistical analysis was performed using Statistical Package for Social Sciences (SPSS) version 20.0 (IBM Corp., Armonk, NY, USA). Quantitative variables were expressed as mean (standard deviation (SD)). Frequencies and percentages were computed for qualitative variables. Two-by-two (2 × 2) tables were constructed in order to calculate sensitivity, specificity, positive predictive value (PPV), negative predictive value (NPV) and overall accuracy of CT scout film and chest X-ray for detection of rib fractures using complete chest CT scan as the gold standard. For the purpose of these calculations, we used the following definitions: (i) true positive—a CT scout film or chest X-ray was said to be true positive if it accurately delineated rib fracture(s) which were also seen on a complete chest CT scan, such as in Figures 1-2; (ii) true negative—a CT scout film or chest X-ray was said to be true negative if it did not reveal any rib fracture and the complete chest CT scan was also negative for rib fracture; (iii) false positive—a CT scout film or chest X-ray was said to be false positive if it showed evidence of a rib fracture, but, no such fracture was noted on complete chest CT scan; (iv) false negative—a CT scout film or chest X-ray was deemed as false negative if it did not show any evidence of a rib fracture, when rib fracture(s) was/were noted on a complete chest CT scan.

Sensitivity was calculated as the ratio of true positives to the total number of patients who had rib fractures on complete chest CT scans. Specificity was calculated as the ratio of true negatives to the total number of patients who did not have any rib fracture on complete chest CT scan. PPV was computed as the number of true positive scout films (or chest X-rays) divided by the total number of positive scout films (or chest X-rays). Likewise, NPV was calculated as the number of true negative scout films (or chest X-rays) divided by the total number of negative scout films (or chest X-rays). Overall accuracy was determined by dividing the sum of true positive and true negative scout films or chest X-rays to the total number of patients included in the study. In order to further validate our findings, susceptibility analysis was performed by calculating the sensitivity of CT scout film and chest X-ray by considering each rib separately (a “rib-level” analysis). This was done to assess the impact of discrepancies in the number and location of fractured ribs on the diagnostic performance of CT scout films and/or chest X-rays. As this analysis did not account for clustering of data (“24 ribs per patient”), specificity and overall accuracy was expected to be exaggerated, and hence, not calculated.

## Results

**Figure 3:**
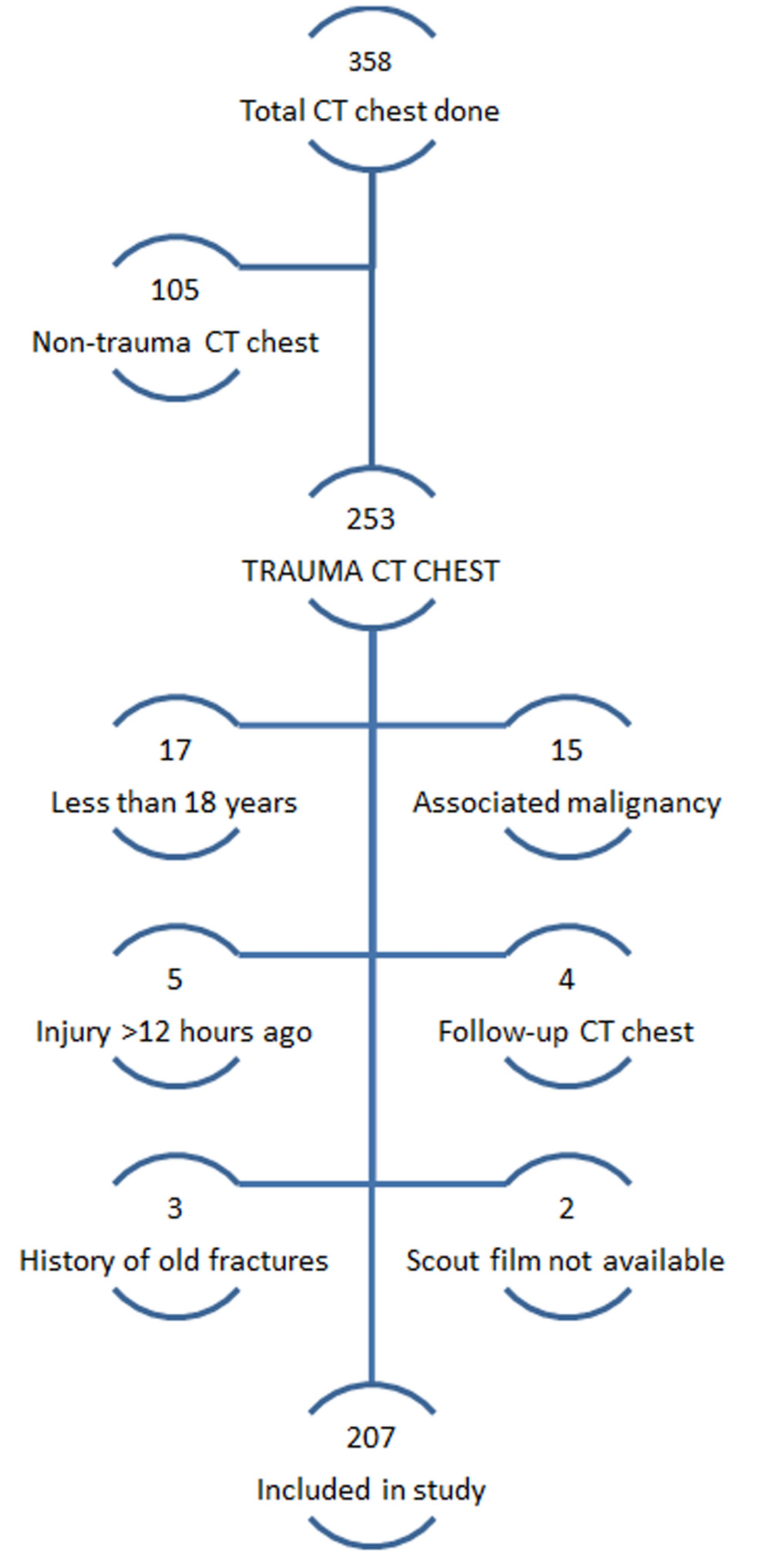
A flow diagram depicting the inclusion and exclusion of subjects in our study

A total of 358 patients underwent CT chest examinations at our institution from October 1, 2013 to September 31, 2014. Among these, 253 patients underwent CT chest for the evaluation of trauma and injuries. After excluding the patients according to the exclusion criteria, a total of 207 patients were included in the study (Figure 3).

Baseline characteristics

**Figure 4:**
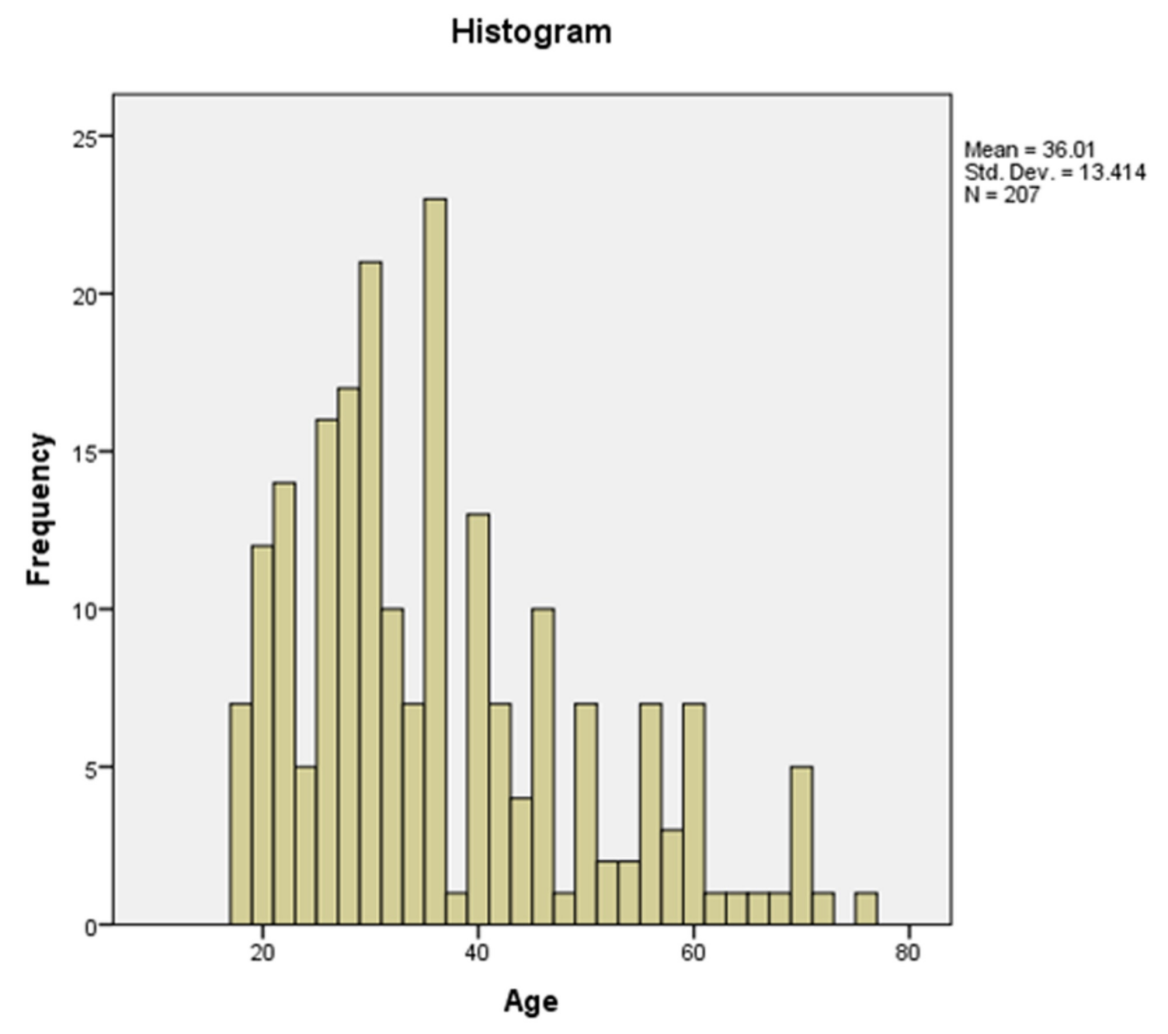
A histogram depicting the frequency of subjects according to various age groups

Mean age of subjects included in the study was 36.0 (SD: 13.4) years with a minimum age of 18 years and maximum age of 77 years (Figure 4). Men constituted the bulk of our sample (n=193, 93.2%). Penetrating and blunt thoracic trauma was reported in 104 (50.2%) and 103 (49.8%) cases respectively. Mechanisms of injury were road traffic accidents (n=96, 46.4%), firearm injuries (n=86, 41.5%), bomb blast injuries (n=14, 6.8%), falls (n=7, 3.4%) and stab wounds (n=4, 1.9%). The average time interval between arrival in the ED and performance of chest X-ray was 35 (SD: 18) minutes. Moreover, the average time between arrival in the ED and performance of chest CT scan was 80 (SD: 37) minutes.

Findings of computed tomography

Rib fractures were noted in 75 (36.2%) subjects. The distribution of rib fractures among patients with blunt and penetrating trauma is given in Table 1. The proportion of men with rib fractures was comparable to the proportion of women who had rib fractures. Other injuries noted on chest CT scans among our patients were pneumothorax (n=102, 49.3%), lung contusion (n=100, 48.3%), intra-abdominal injuries (n=55, 26.6%), hemothorax (n=51, 24.6%), pneumomediastinum (n=18, 8.7%), major vascular injury (n=13, 6.3%), mediastinal hematoma (n=12, 5.8%) and diaphragmatic injury (n=6, 2.9%).

**Table 1:** Frequency of rib fractures among male and female study subjects with penetrating or blunt thoracic trauma

FINDINGS OF COMPUTED TOMOGRAPHY	PENETRATING TRAUMA		BLUNT TRAUMA		Total
	Men	Women	Men	Women	
Rib fracture	33	1	37	4	75
No evidence of fracture	68	2	55	7	132
Total	101	3	92	11	207

Diagnostic performance of scout film

On CT scout film, true positive, true negative, false positive and false negative findings were noted in 42 (20.3%), 116 (56%), 16 (7.7%) and 33 (15.9%) cases, respectively (Table 2). Sensitivity, specificity, PPV, and NPV of CT scout film for detection of rib fractures was 56%, 87.9%, 72.4%, and 77.8% respectively. Positive and negative likelihood ratios were calculated to be 4.6 and 0.5 respectively. Overall diagnostic accuracy of CT scout film was 76.3% using complete chest CT scan as the reference standard. Among patients with false negative CT scout films, findings detected on CT scans (other than rib fractures) were pneumothorax (n=15), pulmonary contusion (n=13), hemothorax (n=7) and intra-abdominal injury (n=4). On a rib-level analysis, true positive, true negative, false positive and false negative findings were noted in 78 (1.6%), 4795 (96.5%), 31 (0.6%), and 64 (1.3%) ribs respectively (Table 3). On a rib-level analysis, the sensitivity of CT scout film was computed to be 54.9%.

**Table 2:** A two-by-two table depicting the results of scout film vis-à-vis those of complete thoracic computed tomography (patient-level analysis) FN = false negative; FP = false positive; TN = true negative; TP = true positive.

		THORACIC COMPUTED TOMOGRAPHY		Total
		Positive	Negative	
SCOUT FILM	Positive	42 (TP)	16 (FP)	58
	Negative	33 (FN)	116 (TN)	149
Total		75	132	207

**Table 3:** A two-by-two table depicting the results of scout film vis-à-vis those of complete thoracic computed tomography (rib-level analysis) FN = false negative; FP = false positive; TN = true negative; TP = true positive.

		THORACIC COMPUTED TOMOGRAPHY		Total
		Positive	Negative	
SCOUT FILM	Positive	78 (TP)	31 (FP)	109
	Negative	64 (FN)	4795 (TN)	4859
Total		142	4826	4968

Diagnostic performance of chest X-ray

On plain chest radiographs, true positive, true negative, false positive and false negative findings were noted in 46 (22.2%), 130 (62.8%), 2 (1%) and 29 (14%) cases, respectively (Table 4). Sensitivity, specificity, PPV and NPV of chest X-ray for detection of rib fractures was 61.3%, 98.5%, 95.8% and 81.8%, respectively. Positive and negative likelihood ratios were calculated to be 40.9 and 0.4 respectively. Overall diagnostic accuracy of chest X-ray was 85% using complete chest CT scan as the reference standard. On a rib-level analysis, true positive, true negative, false positive and false negative findings were noted in 87 (1.7%), 4821 (97%), 5 (0.2%) and 55 (1.1%) ribs respectively (Table 5). On a rib-level analysis, the sensitivity of chest X-ray was calculated to be 61.3%.

**Table 4:** A two-by-two table depicting the results of chest X-ray vis-à-vis those of complete thoracic computed tomography (patient-level analysis) FN = false negative; FP = false positive; TN = true negative; TP = true positive.

		THORACIC COMPUTED TOMOGRAPHY		Total
		Positive	Negative	
CHEST X-RAY	Positive	46 (TP)	2 (FP)	48
	Negative	29 (FN)	130 (TN)	159
Total		75	132	207

**Table 5:** A two-by-two table depicting the results of chest X-ray vis-à-vis those of complete thoracic computed tomography (rib-level analysis) FN = false negative; FP = false positive; TN = true negative; TP = true positive.

		THORACIC COMPUTED TOMOGRAPHY		Total
		Positive	Negative	
CHEST X-RAY	Positive	87 (TP)	5 (FP)	92
	Negative	55 (FN)	4821 (TN)	4876
Total		142	4826	4968

## Discussion

Data regarding the utility of CT scout film for diagnosis of rib fractures and/or other thoracic injuries is virtually non-existent. The value of CT scout film for evaluation of patients with thoracic trauma has remained unexplored hitherto. In this cross-sectional study, we determined the diagnostic accuracy of CT scout film for the detection of rib fractures. We included a sample of 207 patients presenting to the department of radiology of a tertiary care teaching hospital, out of which 75 (36.2%) had rib fractures on chest CT. We observed that CT scout film had a sensitivity and specificity of 56% and 87.9% respectively, which was comparable to that of plain chest radiograph. However, given the low NPV (77.8%) of CT scout film, CT chest still remains the modality of choice for detailed evaluation of thoracic injuries.

Most patients included in this study were young (70.5%) i.e., less than 40 years of age. This trend was similar to previously published studies, which reported a preponderance of younger patients [15]. Nearly all patients included in our sample were male 194 (93.7%), which is in line with previous reports [16]. Male dominance in research literature published from lower middle-income countries in general, and Pakistan in particular, is well-recognized [17]. Few women drive cars and/or motorcycles, and therefore, are less likely to be involved in road traffic accidents as compared to men. Moreover, few women suffer intentional and unintentional injuries as compared to men. Likewise, women are far less likely to suffer firearm injuries as compared to men. All these factors culminate in the gender disparity observed in our sample.

In this study, we observed an almost equal proportion of patients with blunt and penetrating trauma. The most common causes of blunt and penetrating trauma in our study were motor vehicle accidents and firearm injuries respectively. This pattern of injuries was similar to that reported by previously published reports from Pakistan [18] and elsewhere [19]. The overall prevalence of rib fractures in our study sample was 36.2%. Among patients with blunt and penetrating trauma, the prevalence of rib fractures was 39.8% and 32.7% respectively. Our results are in concordance with previous studies, which have also reported a slightly higher prevalence of rib fractures among patients with blunt trauma as compared to those with penetrating trauma [14,20].

Apart from rib fractures, common associated injuries among study subjects included pneumothoraces, pulmonary contusions, hemothoraces, and intra-abdominal injuries. Abdominal visceral injuries were more common among patients who had rib fractures as compared to those without rib fractures. Association of pulmonary contusion, hemothorax, and pneumothorax with rib fractures has been described previously [21]. These associations are important for physicians, surgeons and radiologists as the presence of any one injury on physical examination or initial radiologic imaging should prompt further evaluation. For instance, a diaphragmatic injury was more common among those patients in our study who had fractures of the lower four ribs (eighth to twelfth ribs). These associations are plausible considering the anatomical and physiological basis for these injuries and, from a clinical perspective, can alert doctors to evaluate patients with such injuries more vigilantly.

With respect to diagnostic performance, CT scout film had fair overall accuracy 76.3% in our study. The sensitivity of CT scout film for detection of rib fractures was 56% and specificity of CT scout film was 87.9%. These statistics imply that CT scout film is not useful as a “rule out” test i.e., many patients with a normal CT scout film may have rib fractures on chest CT scan. Conversely, most patients with an abnormal CT scout film will have rib fractures. Given that rib fractures are associated with other injuries, it is imperative that patients with a rib fracture on a CT scout film are evaluated further. Furthermore, NPV of CT scout film was relatively low (77.8%), which may be accounted for by the high prevalence of rib fractures in our study sample. Most patients included in our study had suffered from high-energy blunt or penetrating thoracic trauma, which substantially increases the pre-test probability of a rib fracture and/or any associated injury. In such patients, the value of a negative scout film is extremely limited, not only because of its low sensitivity but also because of the high likelihood of an intra-thoracic injury, which would necessitate a thoracic CT scan per se.

Little data is available regarding the diagnostic accuracy of CT scout film for evaluation of thoracic trauma. In our study, we had 33 (15.9%) false negative and 16 (7.7%) false positive cases. A false positive finding implies that a CT scout film was interpreted to have a rib fracture when the thoracic CT scan was negative. Such findings are plausible given the low signal-to-noise ratio of CT scout films and blurring of images. Motion artifacts occurring due to respiratory movements of the chest wall can also produce an illusion of a rib fracture. Conversely, a false negative finding (the inability to detect a rib fracture when it is present on a chest CT scan) often occurs in cases where a rib fracture is subtle and not displaced, or if the rib shadows are obscured by pleuro-pulmonary pathologies. Such fractures are also frequently missed on plain chest radiographs and can only be reliably diagnosed with a thoracic CT scan [22].

A plain chest radiograph has remained the first line modality for evaluation of thoracic trauma. Portable chest radiographs are a vital tool for rapid evaluation of unstable patients who cannot be transported to the radiology department for comprehensive imaging. Consequently, it is of considerable interest to compare the diagnostic accuracy of plain radiographs vis-à-vis CT scout films. In our study, sensitivity, specificity and overall accuracy of CT scout film for detection of rib fractures was 56%, 87.9%, and 76.3% respectively. Chest X-ray had sensitivity, specificity and overall accuracy of 61.3%, 98.5%, and 85%, respectively. Previously published studies have reported a similar diagnostic accuracy of plain radiograph for detection of rib fractures [9,23-28]. In these studies, sensitivity and specificity of plain radiograph for detection of rib fractures was reported to be 42%–60% and 57%–78% respectively. Based on these estimates, it can be inferred that the diagnostic abilities of CT scout film and plain radiograph are comparable. This implies that plain chest radiographs are as good as CT scout films for detection of rib fractures in patients with a history of blunt or penetrating thoracic trauma. In other words, CT scout films do not provide any additional information or advantage over plain chest X-rays for detection of rib fractures.

While the value of plain chest radiograph and CT scout film for evaluation of rib fractures cannot be over-emphasized, it is important to reckon these radiologic modalities as one single facet of a bigger clinical picture. For all clinicians, it is vital to manage and treat the patient as a whole and to not rely entirely on radiologic imaging. History taking and physical examination remain the primary tools for clinical evaluation of patients. Palpation of chest for detecting areas of tenderness can alert the clinician to the presence of rib fractures and possible internal thoracic injuries. Plain chest radiographs and CT scout films may serve as useful adjuncts for emergency clinicians to supplement their clinical acumen. However, appropriate use of these imaging tools ultimately remains in the hands of their end users (i.e., clinicians). Patients with a history of high-energy thoracic trauma, or those with significant abnormal findings on physical examination, have a high likelihood of having rib fractures and/or associated thoracic injuries. For such patients, a complete thoracic CT scan after initial resuscitation is indispensable and clearly warranted. On the other hand, patients with a history of minor chest trauma and overtly normal examination are unlikely to harbor rib fractures. In such patients, the likelihood of a rib fracture and/or any intra-thoracic injury is very low, and therefore, the yield of a CT scout film and/or chest CT chest scan would be meager. A plain chest radiograph would likely suffice in such cases.

Before making any conclusions from this study, it is important to consider the strengths and limitations of this study. This study was the first of its kind to evaluate the diagnostic accuracy of CT scout film and plain chest radiograph vis-à-vis chest CT scan for detection of rib fractures. Being conducted in a lower middle-income country, this study provides a pragmatic estimate of the diagnostic performance and utility of CT scout film for evaluation of patients with chest trauma. However, it should be noted that in this study, all CT scout films were interpreted by an experienced radiologist, which may have provided higher estimates of diagnostic accuracy. In real emergency scenarios, chest CT scans are often read and interpreted provisionally by trainee radiologists. This is especially true during holidays and after usual hours on working days [29], which may adversely affect diagnostic performance. Moreover, the quality of CT scout film is also dependent on the type of CT scanner installed at a particular institution. In our study, all CT scout films were produced on a 640-slice CT scanner. However, in many institutions, a 32- or 64-slice CT scanner is used for radiologic imaging and this may also adversely affect the diagnostic performance of CT scout film.

The spectrum and pattern of injuries observed in our study sample were in agreement with those reported previously by studies conducted in Pakistan and other developing countries [14]. This helps to strengthen the external validity of this study and allows one to generalize the findings of this study to radiology departments of other hospitals of Pakistan. However, in the present study, only a small number of female and elderly patients were included; this can affect the generalizability of our study findings. Women and elderly patients are known to have a high prevalence of osteoporosis and their bone densities are different from that of young men [30]. Consequently, the diagnostic accuracy of CT scout film for the detection of rib fractures in such patients may be different from that observed in this study. Secondly, in this study, technical factors affecting the quality of CT scout films (such as exposure time, tube voltage and current) were not controlled for. This may have affected the diagnostic accuracy of CT scout films in this study and led to an under-estimation of diagnostic performance. However, the accuracy of CT scout film for the detection of rib fractures in this study was still fair. Moreover, variation in exposure time and tube voltage is natural in real patient-care settings, and therefore, determination of diagnostic performance in such settings is likely to provide a more pragmatic estimate.

## Conclusions

The CT scout film had a fair accuracy for detection of rib fractures, which was comparable to that of a plain chest radiograph. However, CT scout film does not provide any additional information or advantage over a plain chest radiograph. In patients with severe thoracic trauma, CT chest remains the modality of choice for accurate delineation of rib fractures and associated internal injuries.
